# Monitoring Autonomic and Central Nervous System Activity by Permutation Entropy during Short Sojourn in Antarctica

**DOI:** 10.3390/e21090893

**Published:** 2019-09-14

**Authors:** H. Birol Çotuk, Adil Deniz Duru, Şamil Aktaş

**Affiliations:** 1 Department of Sport Health Sciences, Marmara University, 34810 İstanbul, Turkey; deniz.duru@marmara.edu.tr; 2 Department of Underwater and Hyperbaric Medicine, İstanbul University, 34093 İstanbul, Turkey; samilaktas@yahoo.com

**Keywords:** permutation entropy, heart rate variability, electroencephalography, spectral analysis, Antarctica

## Abstract

The aim of this study was to monitor acute response patterns of autonomic and central nervous system activity during an encounter with Antarctica by synchronously recording heart rate variability (HRV) and electroencephalography (EEG). On three different time-points during the two-week sea journey, the EEG and HRV were recorded from nine male scientists who participated in “The First Turkish Antarctic Research Expedition”. The recordings were performed in a relaxed state with the eyes open, eyes closed, and during a space quantity perception test. For the EEG recordings, the wireless 14 channel EPOC-Emotiv device was used, and for the HRV recordings, a Polar heart rate monitor S810i was used. The HRV data were analyzed by time/frequency domain parameters and ordinal pattern statistics. For the EEG data, spectral band power in the conventional frequency bands, as well as permutation entropy values were calculated. Regarding HRV, neither conventional nor permutation entropy calculations produced significant differences for the different journey time-points, but only permutation entropy was able to differentiate between the testing conditions. During the cognitive test, permutation entropy values increased significantly, whereas the conventional HRV parameters did not show any significant differences. In the EEG analysis, the ordinal pattern statistics revealed significant transitions in the course of the sea voyage as permutation entropy values decreased, whereas spectral band power analysis could not detect any significant difference. Permutation entropy analysis was further able to differentiate between the three testing conditions as well between the brain regions. In the conventional spectral band power analysis, alpha band power could separate the three testing conditions and brain regions, and beta band power could only do so for the brain regions. This superiority of permutation entropy in discerning subtle differences in the autonomic and central nervous system’s responses to an overwhelming subjective experience renders it suitable as an analysis tool for biomonitoring in extreme environments.

## 1. Introduction

The monitoring of autonomic nervous system (ANS) and central nervous system (CNS) activity by recording physiological effectors is obligatory during a stay in extreme environments, such as Antarctica and in Space. The potential of acute or chronic psychosocial stressors and environmental contingencies to initiate dysfunctional psycho-physiological processes, and even disabling illnesses demands recognition of early pre-nosological patterns in order to establish salutogenic countermeasures [1].

Monitoring the ANS by heart rate variability (HRV) and the CNS by electroencephalography (EEG) has proved to be suitable for practical applications [2]. However, the potential of such biomonitoring to provide relevant information about structural/functional links between environmental contingencies, psychophysiological impacts, and pathophysiological processes is hampered by the complexity of the neuro-effector integration involved [3]. Furthermore, the algorithmic constructing of the HRV/EEG data analysis and the theoretical framework-based interpretation are not generally agreed upon [4,5]. In this context, spectral analysis is a well-established analysis procedure for both HRV and EEG datasets, and has gained popularity in psychophysiological research. This development is mainly the result of the emergence of concepts which relate specific rhythms of the nervous system to specific functions. For the ANS, the dichotomy of sympathetic/parasympathetic activity is believed to be reflected in the ratio of low-frequency/high-frequency rhythms of the heartbeat’s interval durations [6]. For the CNS, researchers have endowed the classical frequency bands (alpha, beta, delta, theta) with a multitude of functions; for example, task-related active processing of the brain has been proposed to be associated with the inhibition of alpha-band oscillations [7]. The motor system version in this regard is the mu-rhythm switching off in time with movement onset or action observation [8]. Nevertheless, the last decade has especially witnessed an increasing interest in the use of additional analytical tools, mainly originating from the theory of complex systems. For the ANS assessment by cardiovascular variability, this has led to the established use of entropy-based metrics in research [9,10] and even standard software packages [11]. For the brain, the complex system par excellence, spectral analysis has been complemented by the search for hidden information in the EEG using several signal-processing techniques, such as independent or principal component analysis, empirical mode decomposition, and common spatial patterns. In this context, complexity metrics stemming from the information/ergodic theory or dynamical system approach with an emphasis on non-linear transitions do provide novel explanatory power [12,13,14]. In this regard, Permutation Entropy (PE) and related transformations using symbol dynamics [15] have presented a meta-analytical tool, as they can be applied to the datasets independent of the physical/physiological system parameters, and a priori knowledge of the system behavior is not necessary [16]. PE was introduced in 1982 by Bandt & Pompe [17], and since then has gained a reputation in many fields of science with numerous applications and also further developments of the basic method [18,19]. In essence, PE is based on the calculation of the partial fulfilment of the total possible system realizations in phase space by employing Shannon Entropy [20]. As a non-linear parameter, PE can be used as an identifier of the time-series regularity—determinism or regularity of the time-series are denoted with small values of PE, while for random time-series, the normalized PE approaches 1. PE also has the advantage that it is weakly affected by noise, and has been found to be successful in detecting transitions in a variety of datasets, ranging from mathematical models to pathophysiological systems, like epileptic brain dynamics [21]. According to this line of thought, we incorporated PE analysis in the conventional time/frequency domain assessment of HRV and EEG recordings from the 1st Turkish Antarctic Scientific Expedition in 2016. We synchronously monitored the ANS and the CNS during this short sojourn in Antarctica on two weeks of sea travel. The primary aim of the study was to evaluate psychophysiological response patterns during the encounter with Antarctica under the conditions of the particular socio-scientific challenges. A further objective was to explore the difference between PE and standard analytical tools in extracting meaningful information from the EEG/HRV data.

## 2. Materials and Methods

### 2.1. Study Concept

The present study was conducted during “The First Turkish Antarctic Research Expedition” in April 2016. Researchers from seven Turkish universities with diverse specializations collaborated together in this first national scientific encounter to Antarctica. All of the participating scholars had to complete scientific research in their particular academic discipline. The diverse scientific program included marine biodiversity, sea ice observation, marine pollution, geology, and medicine. During the last ship cruise at the dawn of the Antarctic winter (the first two weeks in April) and the beginning of ice expansion, the sea voyage of 13 days began from Ushuaia (Argentina). Then, the vessel crossed the Drake Passage to reach Galindez Island in the Antarctic Peninsula (Vernadsky Station of Ukraine), cruised in the Lemaire Channel, and finally returned to Mar del Plata (Argentina). The weather conditions in the Antarctic Peninsula were variable—the daily temperature changed between 5.4 ${}^{\circ }$C to −4.1 ${}^{\circ }$C, and mean wind speed ranged from a light breeze to a moderate gale (from 6 to 32 knots).

### 2.2. Subjects

All of the participants of the expedition were rigorously screened for adverse medical conditions and their physical health status. Although benign medical conditions were detected in some of the participants, none of these minor health restrictions impeded their participation. No serious health problems were reported during the expedition. All medical examinations were performed under the supervision of the Department of Underwater and Hyperbaric Medicine at the Istanbul Faculty of Medicine according to the National Turkish Polar Research Health Standards [22]. The present study was accepted as a medical part of the scientific expedition and approved by the Ethics Committee of Marmara University in Istanbul. During the cruise, the EEG and HRV from nine volunteer male researchers (age range 27–57 years, body mass index range 24.2–32.2 kg/m${}^{2}$) were recorded synchronously.

### 2.3. Data Collection

**Figure 1:**
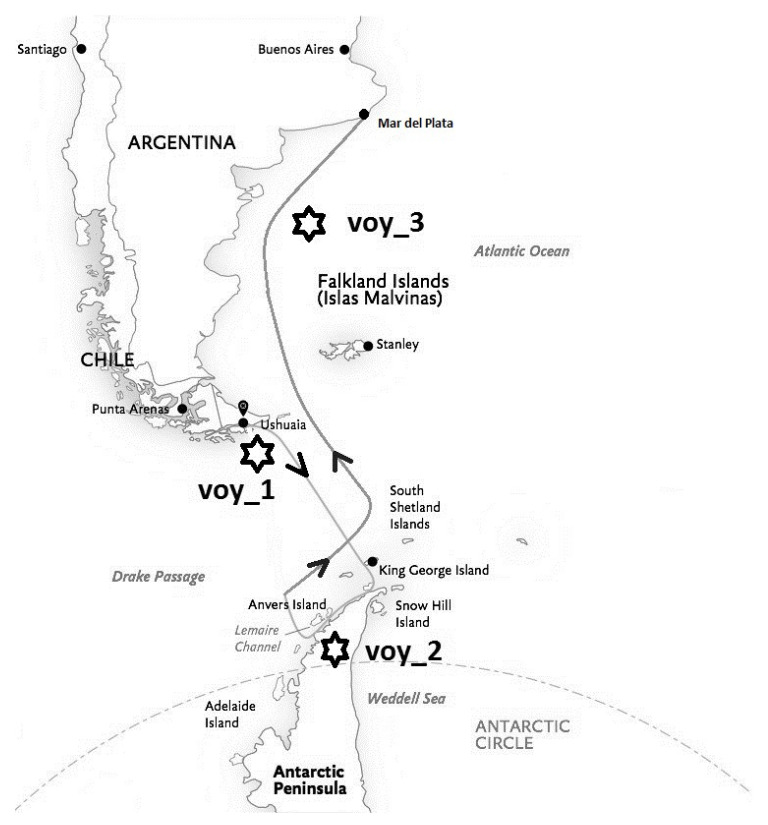
Route of the sea voyage with the three measurement locations (voy).

Three recording sessions were performed on the vessel in a quiet room during calm sea conditions. After leaving Ushuaia harbour, the first recording ($voy_{1}$) was obtained on the second day before crossing the Drake Passage to Antarctica; the second one ($voy_{2}$) on the sixth day in the Antarctic Peninsula (Lemaire Channel, Galindez Island); and the third on the 12th day during the return near Mar del Plata ($voy_{3}$, see Figure 1).

The EEG and HRV recordings were performed in a seated posture and relaxed mental state under three successive conditions; for 5 min with eyes open (EO), for 5 min with eyes closed (EC) and during a 2 min cognitive test (CT) based on space quantity perception. In this CT, the subjects had to repeatedly judge which of two crowdings of randomly distributed dots on the left and right side of the PC screen had more dots [23].

For the EEG recordings, the wireless 14 channel EPOC-$Emotiv^{R}$ EEG device (sampling rate 128 Hz, validity has been shown for brain dynamics research [24]) and for HRV recordings, the $Polar^{R}$ heart rate monitor S810i (sampling rate at 1000 Hz showing excellent agreement with ECG [25]) were used. The same EEG hardware has been successfully used to investigate resting state dynamics of the brain, especially for comparing spectral band activities in the EO/EC conditions [26]. Studies with analogous experimental designs have employed for EEG recordings with minimal epoch durations of 2–3 min for analysis of spectral components, as well as multimodal neuroimaging [27,28].

### 2.4. Data Analysis of HRV

**Figure 2:**
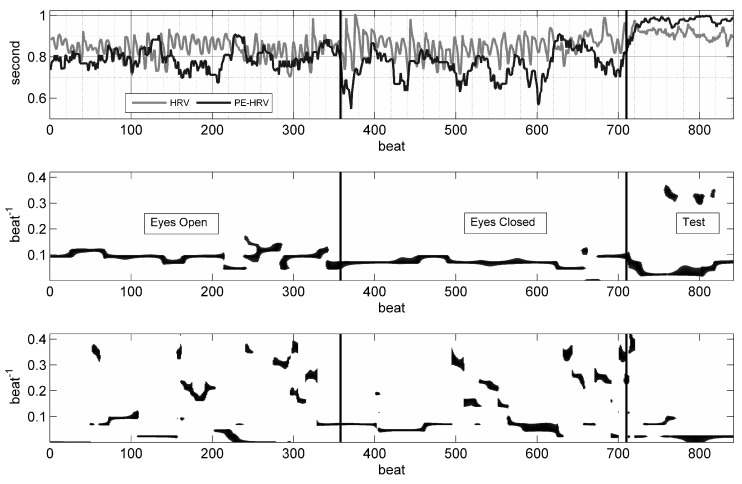
Heart rate variability (HRV) and permutation entropy (PE) time-series with time-frequency distributions (TFD) of HRV and PE. In the upper panel, beat-to-beat intervals (light line) and the PE (D3) time-series (dark line) are shown; note the rhythmic evolution of the PE time-series. During the cognitive test, the RR interval duration became longer, but the fluctuations reduced and became more erratic, which is reflected in the clear increase of PE. In the middle panel, the TFD of HRV shows a continuous rhythm with a period of 10 beats; during the cognitive test, faster rhythms with a period of 3 beats also emerged. In the lower panel, the TFD of PE shows a mixture of slow and fast rhythms.

Each HRV recording was checked for artifacts and non-normal intervals—three supraventricular extrasystoles were detected and corrected in only one subject. The HRV data were analyzed in the time domain by the mean RR interval (RRmean), the root mean square of the differences of successive RR intervals (RMSSD), and the percentage of interval differences of successive RR intervals greater than 50 ms (pNN50). Regarding the frequency domain analysis of HRV, the general agreement on the requirement to provide at least a 5 min recording duration was not met by the CT (the duration was only 2 min) [29]. As for the HF component, this standard could be ameliorated because as a heuristic guideline, a sampling period containing 10 complete cycles of the lowest observed frequency may be regarded as containing sufficient information [30]. Therefore, we calculated only the relative power of the HF component of HRV: HF (%) = HF (ms${}^{2}$)/total power (ms${}^{2}$) × 100%. For each recording epoch (EO, EC, and CT), these HRV indices were calculated separately using the software, Kubios 3.0. The PE calculation of HRV is based on beat-to-beat ordinal patterns. In this naturalistic approach, the various increase/decrease patterns of successive heartbeat intervals are counted—then, the Shannon entropy is computed based on all probabilities for a given embedding (permutation) dimension (for details, see [15]). For each recording epoch, PE was obtained separately by using an embedding dimension 3 (D3) in a sliding window (L) of 30 heartbeats, and τ=1 heartbeat (time delay τ). As D3 allowed for six permutations (3! motifs) and the length of data (L) used for calculation should be at least 5×3! [31], we had to use at least 6×5=30 heart beats for L. For comparison, the same procedure was repeated with dimension 4 (24 motifs) in L=120 heartbeats (5×4!=120), again with τ=1. The correlation coefficient of the PE values for D3 and D4 was 0.892 (p=0.000). Changing the time delay from 1 up to 10 had been attempted in the literature, and thereby the results have inevitably changed [32,33]. The point is that the rhythms inherent in HRV will also be present in the PE time-series, and will non-linearly distort the PE values according to this rhythmic change (see Figure 2). Therefore, we constructed the PE time-series with time delay τ=1 and calculated the mean value for each experimental epoch.

### 2.5. Data Analysis of EEG

The raw EEG data were screened for artifacts and only artifact-free recording parts were subjected to further analysis. Spectral analysis was performed in the conventional frequency bands as delta (0–4 Hz), theta (4–8 Hz), alpha (8–13 Hz), and beta (13–30 Hz). For each condition (EO, EC, and CT) of the experimental paradigm, PE was computed for an embedding dimension 3 and τ=1 in non-overlapping successive time windows having a width of 20 seconds (for parameter choice [34]). Then, the PE values obtained for these time windows were averaged for each condition. The electrodes were grouped into four groups as left (LEg), right (REg), frontal (FRg), and parieto-occipital (POg). FRg was constituted by “AF3, AF4, F3, F4”, POg by “O1, O2, P7, P8”, LEg by “AF3, F7, F3, FC5, T7, P7,O1”, and REg by “O2, P8, T8, FC6, F4, F8, AF4”. The PE values of the electrodes were averaged and assigned to each electrode group. In the second step of the PE analysis, the raw EEG was bandpass-filtered. The filtering process was adopted to compute the time-series having the conventional frequency band contents as delta, theta, alpha, and beta. For each of the resulting time-series, the PE values were computed as described above for the raw EEG.

### 2.6. Performance Analysis in the Cognitive Test

The performance of the subjects in the CT was calculated according to the number of correct judgments (dotN), the ratio of correct judgments to total attempts (dotR), and a composite performance indicator (dotP) incorporating the “dotNduration/testduration” ratio (dotT).

(1) dotP=dotN∗dotR∗dotT

### 2.7. Statistical Analysis

For the HRV indices, a Repeated Measures ANOVA (RMAnova) was computed with the main factors of voyage measurement time and experiment condition. For the EEG indices, RMAnova was computed using the PE or spectral power values with the main factors of measurement time, electrode group, and cognitive task. For the performance values in the cognitive test, RMAnova was computed using the only main factor of voyage measurement time. The Mauchly’s tests for sphericity were used, and Greenhouse-Geiser or Huynh-Feldt results were reported based on epsilon values when the violation of the sphericity was observed. A Bonferroni correction was performed for the post hoc tests, and statistically significant results were reported throughout the study as p<0.05.

## 3. Results

### 3.1. Performance in the Space Quantity Perception Test

The performance in the dot judgment test shown in Table 1 increased during the voyage, but the differences did not reach statistical significance (p>0.05).

### 3.2. Results of the HRV Analysis

In the case of time and frequency domain parameters of HRV (see Table 2), the RMAnova statistics did not show any significant main effects, and neither for voyage time (voy) (p>0.05) nor cognitive condition (cog) (p>0.05). For the PE_3 values of HRV, the RMAnova statistics only yielded significant main effects for the cognitive condition (F(2,16)=12.78,p=0.014). The post hoc tests of the cognitive condition revealed that the PE_3 value of CT was significantly higher than the PE_3 value of EC and EO (MD=0.042,p=0.006;MD=0.042,p=0.003). For the PE_4 values of HRV, the RMAnova statistics only yielded significant main effects for the cognitive condition (F(2,16)=10.09,p=0.043). The post hoc tests of the cognitive condition revealed that the PE_4 value of CT was significantly higher than the PE_4 value of EC and EO (MD=0.047,p=0.012;MD=0.045,p=0.009).

### 3.3. Results of the EEG Analysis

#### 3.3.1. Power Spectrum Analysis of EEG

In the case of delta and theta band power spectrum values, the RMAnova statistics showed no significant main effects for voyage time (voy) (*p* > 0.05), cognitive task (cog) (*p* > 0.05), and brain region (brg) (*p* > 0.05). For alpha band power spectrum values, the RMAnova statistics yielded significant main effects for brain regions (F(3,24)=4.65,p=0.011) and the cognitive task (F(2,16)=4.95,p=0.021). When the post hoc tests of the brain regions were computed, the right brain region alpha band power was found to be higher than the left brain region (MD=2.88 μV, *p* = 0.031). In addition to this, the post hoc tests of the cognitive condition revealed significant alpha band power differences between EC and EO (MD=2.67 μV, *p* = 0.006). When the beta band power spectrum values were investigated with RMAnova, a significant main effect only for the brain regions (F(3,24)=3.57,p=0.029) was observed. Beta band power of the right brain region was found to be higher than the left brain region (MD=3.25 μV, p=0.05).

#### 3.3.2. PE Analysis of Raw EEG

In the Repeated Measures Anova statistics, there were significant main effects for voyage time (voy) (F(2,16)=3.79,p=0.045), cognitive condition (cog) (F(2,16)=18.83,p<0.001), and brain region (brg) (F(3,24)=7.16,p=0.001). The average PE value of the first voyage recording was found to be greater than the mean PE value of the third voyage recording (MD=0.014,p=0.05). The mean PE values of the voyages have been summarized in Table 3.

For the cognitive condition, the PE values of EO and CT were found to be larger than the PE value of EC (MD=0.022,p=0.002;MD=0.026,p=0.006).

#### 3.3.3. PE Analysis of EEG Frequency Bands

In the RMAnova statistics, PE values of the delta band yielded significant main effects for the brain region (F(3,24)=23.33,p<0.001) and cognitive task (F(2,16)=15.72,p<0.001). The right brain region PE value of the delta band was found to be higher than the left (MD=0.009,p<0.001). When the post hoc tests for the cognitive task were computed, the PE value of the delta band of EC was found to be higher than the values of EO and CT (MD=0.019,p=0.006,MD=0.02,p=0.014), and this effect was observable in each brain region as a significant interaction between the brain region and cognitive condition (F(2.24,17.92)=7.16,p=0.004). In the RMAnova statistics, PE values of the theta band yielded significant main effects for the brain region (F(3,24)=25.82,p<0.001) and cognitive task (F(2,16)=33.06,p<0.001). The post hoc tests revealed that the average PE value of the PO brain region was greater than the PE values of the other brain regions (p<0.05 for each comparison). When the post hoc tests for the cognitive condition were computed, the PE value of the theta band of EC was found to be higher than the values of EO and CT (MD=0.012,p=0.003,MD=0.018, p=0.001), and the PE value of EO was observed to be significantly greater than the corresponding value of CT (MD=0.006,p=0.004). In the RMAnova statistics, PE values of the alpha band yielded significant main effects for voyage times (F(2,16)=9.76,p<0.002) and cognitive condition (F(2,16)=31.40,p<0.001). The post hoc tests revealed that the PE value of the alpha band at the first voyage point was significantly higher than the third voyage point (MD=0.005,p=0.008). According to the post hoc tests of the cognitive condition, the PE value of the alpha band for EC was observed to be lower than the EO and CT tasks (MD=−0.015,p=0.001,MD=−0.018,p=0.001). Moreover, there was a significant interaction between the cognitive task and the brain regions. During the EO and CT conditions, the PE value of the alpha band of PO was shown to be higher than that of the frontal region (MD=0.004,p=0.003,MD=0.004,p=0.018). In the RMAnova statistics, PE values of the beta band yielded significant main effects for the brain region (F(3,24)=19.95,p<0.001) and cognitive task (F(2,16)=13.79,p<0.001). The left brain region was shown to have a higher PE value in the beta band than the right brain region (MD=0.006,p=0.005). The post hoc tests of the cognitive condition revealed that the PE value in the beta band of EC was significantly lower than both EO and CT (MD=−0.009,p=0.008;MD=−0.012,p=0.012).

## 4. Discussion

This study was the first attempt to synchronously record HRV and EEG in order to evaluate ANS and CNS response patterns during a confrontation with the Antarctic habitat on a two-week sea voyage. In a few studies conducted in Antarctica, either HRV or EEG have been used, but not both together. Additionally, by applying ordinal pattern statistics to the EEG and HRV time-series, a subtler view of the autonomic and central nervous system processes could be compiled, rather than by only using spectral and time-domain methods.

### 4.1. Evaluation of the HRV Dynamics

HRV was recorded for visitors of Antarctica in only two studies, and the analysis mostly relied on spectral parameters. Farrace et al. [35] measured HRV and various hormones before, at the beginning, and after a 40−day stay in Antarctica. They reported significant decreases in the measured anterior pituitary and adrenal hormonal levels, and found a decrease in the LF power as the sole changing parameter of HRV. They concluded that, although being relatively short, the exposure to the Antarctic environment induced a general psycho-physiological trophotropic response. Similarly, Harinath et al. [36] monitored the members of an Indian Antarctic summer expedition with a duration of 60 days. They reported that the LF power of HRV, as well as the urinary excretion of epinephrine, norepinephrine, and salivary cortisol increased on day 7 of the stay and returned to the starting Delhi values by day 60. The authors suggested that this short Antarctic residency during an austral summer resulted in gradual attenuation of the initially elevated sympathetic tone and a shift of the ANS activity toward the parasympathetic mode. Interestingly, in the other group who had stayed over winter in Antarctica, the sympathetic nervous system activity had risen. The conclusions of both studies relied on the proposed close link between LF power and sympathetic nervous system activation, but this assumption has been substantially challenged. In-depth analysis of published research has shown that the LF component of the HRV spectrum is predominantly determined by vagal activity and baroreflex control [37].

The findings of our study do not support such changes in the activity of the ANS subsystems during this short sojourn in Antarctica. The time and spectral domain parameters, as well as the PE variable of HRV, remained stable throughout the sea voyage. However, PE was the sole indicator of changing heartbeat dynamics when the subjects engaged in the space quantity perception test, and this finding was replicable in all three recordings. This finding emphasises the notion that PE analysis of short HRV recordings has the potential to outweigh time and frequency domain HRV parameters in monitoring psycho-physiological transitions [21,38]. In this particular case of HRV monitoring, PE analysis was able to capture the transition to a more irregular and constricted heartbeat interval sequence (see Figure 2), whereas the time and frequency domain analysis provided only slight and non-significant hints. Although spectral indices of HRV, like the LF/HF ratio, are generally regarded to reflect autonomic balance, the critique of these spectral parameters questioned the validity of this approach [4,39]. Mentally challenging tasks with the aim to increase mental effort/load mostly resulted in reductions of both HF and LF oscillations [40]. Such lowering of HRV has been consistently shown during cognitive activities with demanding attentional and perceptual resources [41]. The space quantity perception test used in this study was initially developed for the Navy, and challenges the subjects by using perceptual difficulty and sustained attention. Thus, the lower HRV during the CT was expected, but not the fact that this transition was recognized solely by the PE analysis. Albeit non-significant, the performance in the dot judgment test increased during the voyage, which may have been due to a learning effect. However, more importantly, there was no cognitive performance decrement and no change in the reaction pattern of HRV.

An analogous finding was reported for emergency physicians in the natural setting of their daily routine of pre-hospital patient care. Cognitive workload transitions were best assessed by the changes in PE values of HRV, whereas other HRV indices were less informative [42]. Similarly, PE was more discriminate for the transitions of HRV of anesthetists between the anesthesia stages of “induction” and “maintenance” [43]. PE analysis of short HRV data was also able to differentiate between basic emotional states: Compared to neutral emotional states, PE values increased significantly during the emotional states of happiness, sadness, anger, and disgust [33].

In the clinical realm, PE analysis of HRV complexity in diabetic cardiovascular autonomic neuropathy was able to discriminate patients from healthy controls. The decrease in HRV complexity provided additional information to that obtained with traditional HRV methods. Using Dimensions 3 and 4 provided similar results, but the correlation coefficients with the classical time and frequency domain parameters of HRV were low and mostly non-significant [32]. Graff et al. [44] tried to differentiate vasovagal syncope patients by examining time-domain and entropy-based HRV parameters recorded in advance to the head-up tilt testing. During spontaneous breathing, the PE of HRV did not provide any significant information for the differentiation between fainters and non-fainters, but was the sole significant HRV parameter when the respiration rate was controlled.

### 4.2. Evaluation of the EEG Dynamics

The rare studies employing EEG recordings in Antarctica have been exclusively conducted during overwinter stays, and data analysis performed by spectral analysis. The first study in this regard revealed significant changes in the EEG beta-band as stronger desynchronization and an accompanying enhancement of neurasthenic symptoms with longer stay [45]. In a more recent study, EEG was recorded in eight male volunteers isolated during the Antarctic winter period, while cognitive tests and affective state questionnaires were applied in relation to exercise logs. Regularly physically active people showed steady mood and decreased brain activity (both in the alpha and beta bands) in the course of isolation. Brain activity in the alpha and beta bands of inactive people instead first increased and then remained high, accompanied by a deterioration of mood. This indicates a constant/elevated level of cortical arousal of the inactive subjects in response to the monotony of an over-winter stay in Antarctica. No effect of exercise and isolation on cognitive performance was found, which increased non-significantly [46].

**Figure 3:**
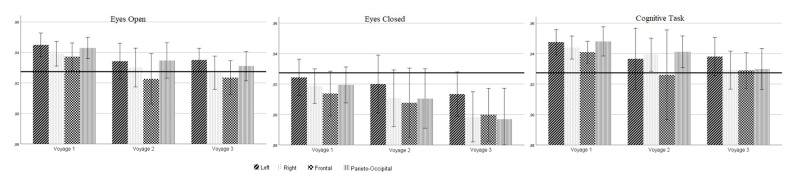
PE values of the EEG according to measurement time, cognitive condition, and brain region with grand mean.

The striking finding in the present study is the ability of PE analysis to detect significant differences in the complexity/regularity of EEG during the voyage where conventional spectral analysis failed. The difference of PE values was significant between the first and third measurement, which means that for the whole brain dynamics, the PE analysis distinguished between the beginning and end of the voyage, whereas spectral analysis did not ($voy_{1}$ versus $voy_{3}$, see Figure 1). Albeit non-significant, the PE values of the second measurement after reaching the Antarctic Peninsula were in-between (Figure 3), thus the EEG became more regular throughout the voyage. Regarding EEG spectral power, none of the four classical spectral bands was able to track this transition. However, when these spectral EEG bands were separately subjected to PE analysis, a significant increase of regularity (PE decrease) between the first and last recording in the alpha band could be replicated (but not in the other frequency bands). By assessing increases/decreases in the irregularity of the EEG, dimensional or symbolic complexity indices may indicate an enhanced/lowered distress level better than spectral power values do [47]. In this context, PE analysis of the EEG has proven to be effective in discriminating between calmness and distress [48,49].

Together, these EEG findings denote a stepwise calming down of the subjects throughout the voyage. Although we did not collect other psychometric indices during the expedition, the unique scientific–sociological context of the journey possesses explanatory power for these psychophysiological findings. Although Turkey joined the Antarctic Treaty in 1995, only in 2016 was an initiative of 13 researchers from 7 universities able to carry through the first Antarctic expedition of Turkey. Besides the huge media awareness and outreach activities, the Turkish scientific community focused on this inaugurate expedition. That had an enormous physio-psycho-social impact on the researchers, because all had to complete a scientific study (with hopefully good results) in a very limited time. Fortunately, the outcome of the expedition was unexpectedly successful as high-quality scientific reports were published, besides discovering a new fungus which was named after the scientific leader of the expedition [50]. Moreover, due to this success, the government took over the responsibility for further Antarctic expeditions as state-governed activities, and three further Antarctica expeditions have been carried out to date in that governance. In this special social setting, the first EEG and HRV recording of the voyage was performed in a mental state of anticipation, with the researchers awaiting the encounter and first impression of Antarctica and hoping for the fortune of their specific research. The second recording was done under the influence of the elusive beauty of Antarctica but also on the ongoing duties of the individual research activities. The third recording was accomplished during an almost completed return journey in a state of mind characterized by a belief in largely meeting the individual and scientific goals.

When the cognitive conditions were compared, the PE indexed complexity increased during the EO and CT tasks when compared with the EC resting period. In the frequency domain, only the alpha spectral band power exhibited significant changes between the cognitive conditions, the EC resting period displaying the greatest spectral power. This finding was also reflected in PE value changes of the individual alpha band, where the PE values of EC were significantly lower than those of the EO and CT tasks. This can be conveniently explained by how when closing the eyes, the dominant alpha rhythm creates a more “ordered” brain state, which becomes more “complex” as more brain regions are desynchronized while the brain transitions to an eyes-open task-performing state [7]. Further breaking down the PE analysis to the other frequency bands revealed that the PE values of the beta band displayed analogous significant transitions. Contrary to this, the PE values of the delta and theta bands in the EC resting state were significantly higher than in the task-related EO and CT conditions. It has been shown that in the frequency domain, alpha and theta power changed antagonistically in response to mental workload elevations, where alpha power decreases and theta power increases. Enhancement of task complexity and heightened demand for attentional resources accentuates this switch [51,52]. The present study findings denote that in the lower frequency range of brain waves (<8 Hz), the eyes-closed condition could be characterized as “lower-ordered”, but on the contrary, as “higher-ordered” in the higher frequency range (8–30 Hz). In the frequency domain, only alpha band power could recognize the coarse transition to eye closure, whereas PE analysis based on the regularity/irregularity dichotomy was able to detect the subtle task-oriented functional brain dynamics within each particular frequency band.

Regarding the differences between brain regions, in the frequency domain, only alpha and beta band power of the right brain region was found to be significantly higher than in the left brain region. In the beta band, PE values of the right brain region were lower than for the left brain region. Thus, throughout the recordings, the right brain presented as “less desynchronized” or “more ordered” activity in the beta band, which translated into lower complexity and stronger spectral power of the beta band. Such hemispheric asymmetries have been related to types of cognitive control processes and creativity [53,54], which may be relevant in this particular case since the participants were all successful academicians.

PE analysis of EEG data has been successfully implemented in clinical research to monitor anesthesia depth [55] or monitor seizure activity in epilepsy [56]. In the physiological context, PE was employed to classify sleep stages [57] or evaluate the complexity of the healthy EEG [20]. The common denominator of these studies and our findings is the superior ability of PE analysis to extract relevant hidden information from the otherwise not accessible microstructure of the EEG.

### 4.3. Limitations

In the present study, we had no opportunity to obtain baseline recordings both for HRV and EEG before the start of the expedition, as the participating subjects had been living in different cities and joined the team just on the departure day in Istanbul. However, after flight travel and arrival in Ushuaia, there was sufficient time for recovery and night rest. The sea journey started at late evening of the next day with calm sea conditions, which again provided quiet night rest. Actually, the first recordings were obtained on the first morning after short sea travel in proximity to the South American coast. Thus, sufficient physical regeneration and sleep, combined with the then uniform and calm housing environment substituted a “baseline” by a “standardized starting” recording. Regarding the psycho-physiological adaptation processes, both types of recordings (baseline and standardized starting) could be considered equally valuable for comparison with later time-points. Relatedly, we recognize that the lack of structured qualitative and quantitative psychometric data limits our assessment of the effective psycho-social contingencies. Therefore, we have only reported on the overarching and common themes acknowledged by all participants, which we evaluated in extensive interviews before the expedition and on the measurement days. One further limitation of the present study is the small number of subjects participating in the study. Unfortunately, this is a common denominator of almost all medical and psychophysiological studies in extreme environments, particularly those in Antarctica. Although this classic critic is obviously valid, the need for expanding knowledge on human adaptation to extreme conditions best illustrated by the challenges of spaceflight warrants new methodological approaches, which should preferably be tested in Antarctica [58]. Therefore, we employed the mathematical tool PE under rigorous statistical constraints by using parametric statistics in such a small sample size. The present outcome in favor of PE turns out as the strength of ordinal pattern statistics in identifying ”micro-effects” in physiological data contingent on the psycho-ecological state of the subject.

## 5. Conclusions

Ordinal pattern-based complexity measures bears the potential to enrich time-series analysis in detecting transitions or patterns in empirical data which may be overlooked by other analytical tools. This has been keenly demonstrated for PE in the present HRV and EEG data from the peculiar Antarctic sojourn. Care should be taken not to over-interpret this approach, since applying PE of dimension m=3 and time delay τ=1 to the datasets may only estimate the centroid of the resulting weighted power spectrum. Thus, this special parameter constellation might only be considered as a “spectral estimator in disguise” [59]. In addition, the use of higher time delays may especially change the results as the rhythms inherent in the data structure will inevitably distort the ordinal patterns. Similarly, coarse-graining procedures which employ sub-sampling of the data set may not adequately reflect the real (non-linear) nature of the physiological process under investigation. Nevertheless, PE has the major advantage of being robust against noise, as outliers are weighted not much differently than the other values.

Both the challenges and advantages of PE should be tested by: (i) Extending the ordinal pattern statistics to the spatial domain, where analysis of the dynamical complexity of spatio-temporal systems by PE may be effective in large datasets [60]; (ii) Assessment of information transfer and coupling between time-series by PE and derived analytical tools [61,62], especially addressing the direction of coupling [63]; (iii) Examination of the effectiveness of ordinal pattern statistics in detecting non-linear transitions and pattern changes in time-series [21,38], especially for practical applications [64]; and (iv) Machine learning and data classification applications using deep learning algorithms [65].

## Figures and Tables

**Table 1:** The results of the dot judgment test. Results are shown as μ±σ(p>0.05).

	${\mathrm{voy}}_{1}$	${\mathrm{voy}}_{2}$	${\mathrm{voy}}_{3}$
dotN	42.3 ± 6.5	46.3 ± 4	49.3 ± 6.6
dotR (%)	71.2 ± 3.7	73.5 ± 5.4	76.0 ± 7.1
dotP	15.8 ± 2.2	17.8 ± 2.7	19.4 ± 4.5

**Table 2:** HRV indices. Results are shown as mean ± standard deviation (* denotes p<0.05).

	${\mathrm{voy}}_{1}$			${\mathrm{voy}}_{2}$			${\mathrm{voy}}_{3}$		
	**EO**	**EC**	**CT**	**EO**	**EC**	**CT**	**EO**	**EC**	**CT**
Rrmean (ms)	770±190	779±195	793±195	817±149	818±141	829±148	795±142	796±144	811±140
RMSSD (ms)	19±8.6	20.6±10.6	17.0±6.8	21.7±8.5	20.5±7.3	20.6±6.6	20.2±9.9	19.7±9.4	18.9±7.3
pNN50 (%)	2.2±3.0	3.6±4.9	1.1±2.7	3.4±6.3	2.6±5.0	1.9±3.9	3.8±6.6	3.7±6.1	1.9±3.7
%HF	16.6±8.5	19.2±12.1	21.5±21.6	20.7±11.0	17.2±8.4	19.9±16.7	16.7±7.9	18.6±13.8	19.4±11.0
PE_3	0.88±0.05	0.88±0.05	0.92±0.04 *	0.88±0.05	0.89±0.05	0.93±0.03 *	0.90±0.04	0.89±0.03	0.93±0.02 *
PE_4	0.84±0.07	0.84±0.07	0.88±0.04 *	0.84±0.07	0.84±0.06	0.89±0.05 *	0.86±0.05	0.86±0.04	0.91±0.03 *

**Table 3:** Permutation entropy (PE) values of raw electroencephalography (EEG).

Voyage No	Mean	Std. Error
1	0.935	0.003
2	0.926	0.006
3	0.921	0.005
